# Breathe In, Breathe Out: Metabolic Regulation of Lung Macrophages in Host Defense Against Bacterial Infection

**DOI:** 10.3389/fcimb.2022.934460

**Published:** 2022-07-08

**Authors:** J. Tucker Andrews, Daniel E. Voth, Stanley Ching-Cheng Huang, Lu Huang

**Affiliations:** ^1^ Department of Microbiology and Immunology, University of Arkansas for Medical Sciences, Little Rock, AR, United States; ^2^ Department of Pathology, Case Western Reserve University School of Medicine, Cleveland, OH, United States; ^3^ Case Comprehensive Cancer Center, Case Western Reserve University School of Medicine, Cleveland, OH, United States

**Keywords:** immunometabolism, lung macrophages, bacterial infection, trained immunity, inflammation

## Abstract

Lung macrophages are substantially distinct from other tissue-resident macrophages. They act as frontier sentinels of the alveolar-blood interface and are constantly exposed to various pathogens. Additionally, they precisely regulate immune responses under homeostatic and pathological conditions to curtail tissue damage while containing respiratory infections. As a highly heterogeneous population, the phenotypes and functions of lung macrophages with differing developmental ontogenies are linked to both intrinsic and extrinsic metabolic processes. Importantly, targeting these metabolic pathways greatly impacts macrophage functions, which in turn leads to different disease outcomes in the lung. In this review, we will discuss underlying metabolic regulation of lung macrophage subsets and how metabolic circuits, together with epigenetic modifications, dictate lung macrophage function during bacterial infection.

## Introduction

Macrophages are renowned for tightly regulating multiple functions *via* metabolic activities. Many fundamental discoveries in macrophage immunometabolism were made by studying *in vitro*-polarized classically- (M1) and alternatively- (M2) activated macrophages, also known as the M1 and M2 paradigm of macrophage activation (O’neill et al., 2016; Van Teijlingen Bakker and Pearce, 2020). Briefly, M1 macrophages use glycolysis for ATP generation that supports production of pro-inflammatory cytokines. In contrast, M2 macrophages largely rely on mitochondrial oxidative phosphorylation (OXPHOS) activity fueled by fatty acid oxidation (FAO) and glucose oxidation. This area has been reviewed extensively elsewhere (Pearce and Pearce, 2013; O’neill et al., 2016; O’neill and Pearce, 2016). While the M1 and M2 paradigm has provided greater understanding of macrophage biology, this classification has no doubt oversimplified the actual complexity of macrophage phenotypes and fails to fully recapitulate immunological functions of tissue-resident macrophages in homeostasis and during *in vivo* infection. Macrophages in the lung function as key regulators of host defense and homeostasis. As the most abundant immune cells in the lung under homeostatic conditions, pulmonary macrophages are distinct from macrophages in other tissues due to their constant exposure to foreign particles and various respiratory pathogens. Therefore, these cells must employ unique strategies to minimize and resolve tissue damage induced by inflammatory responses, while eliminating and/or tolerating insults, such as pathogens and pathogen products. For instance, compared to other tissue-resident macrophages, alveolar macrophages (AMs), the major macrophage population in the lung alveolar space, are less able to initiate effective immune responses as they express low levels of MHCII, but high levels of regulatory molecules such as CD200R and CD172α (Hussell and Bell, 2014). On the other hand, interstitial macrophages (IMs), located in the lung interstitium, express different cell surface proteins and have distinct transcriptional profiles (Hussell and Bell, 2014; Gibbings et al., 2017; Leach et al., 2020). In this review, we will discuss recent findings regarding the metabolic reprogramming in lung macrophages during bacterial infection and highlight cellular metabolic pathways that influence infection outcomes.

## Alveolar Macrophages and Mitochondrial Metabolism

**Figure 1:**
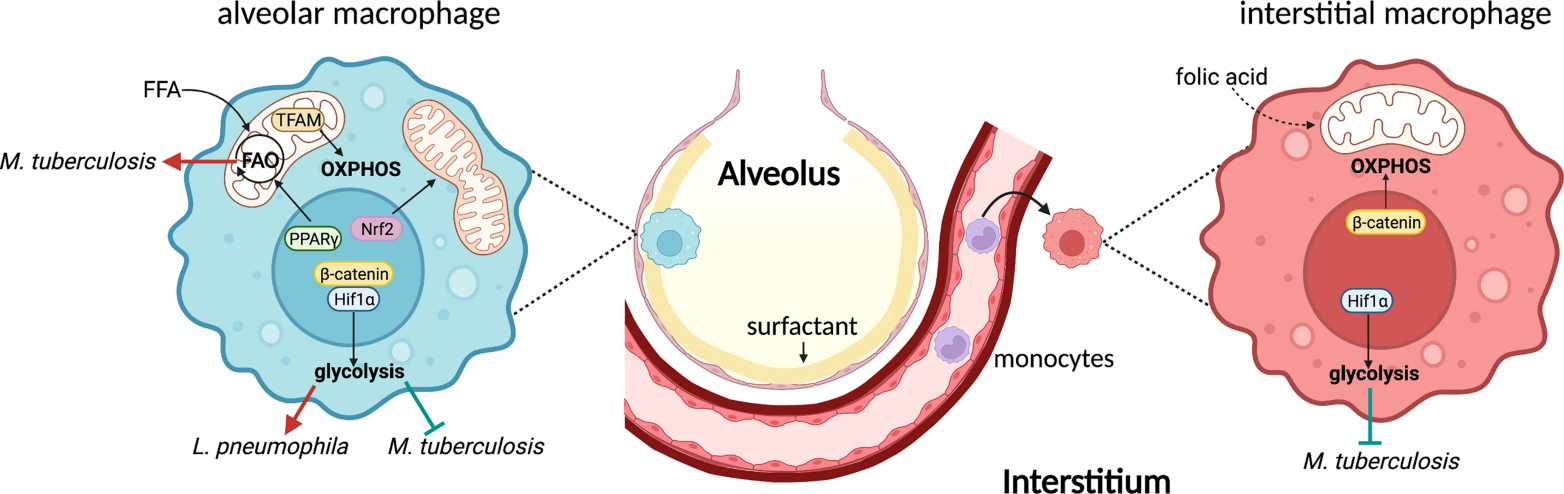
Lung macrophages exhibit distinguished metabolic pathways during bacterial infections. AMs reside in the alveolar space and express high level of PPARγ to promote FFA uptake and FAO. Nrf2 activation leads to mitochondrial fusion which in turn promotes OXPHOS in AMs. Additionally, TRAM-mediated mitochondrial metabolism is essential for AM maintenance and function. Hif1α is the key transcription factor to promote glycolysis in AMs. β-catenin is required for this process, at least during viral infection. AMs represent a permissive cellular niche for Mtb, likely because of the high FAO. Glycolysis inhibits intracellular Mtb growth, whereas it promotes *L. pneumophila* replication. On the other hand, IMs originate from monocytes and control Mtb infection, largely due to the high glycolytic activity mediated by Hif1α. β-catenin, however, promotes mitochondrial respiration in IMs. One subset of IMs expresses folate receptor, an indication of their capability to utilizing folic acid. FFA, free fatty acids; FAO, fatty acid oxidation.

AMs are embryonically-derived and mainly arise from yolk sac and fetal monocytes (Guilliams et al., 2013; Gomez Perdiguero et al., 2015). Due to the uniqueness of their location, AMs are soaked in fluid that contains high levels of surfactant. Pulmonary surfactant, produced by type-II alveolar cells, is composed of phospholipids and surfactant proteins (Trapnell et al., 2003). To adapt to this lipid-rich environment, AMs express high levels of peroxisome proliferator-activated receptor γ (PPARγ), a critical transcription factor that regulates expression of genes involved in lipid metabolism to catabolize surfactant (Baker et al., 2010) (**Figure 1**). Mitochondria, the central hub of cellular signaling pathways, function as the master regulators for energy metabolism and effector functions in AMs under various conditions. Murine AMs exhibit higher OXPHOS but lower glycolysis compared to bone marrow-derived macrophages (BMDMs) and peritoneal macrophages in steady state (Svedberg et al., 2019; Woods et al., 2020). A recent study further demonstrated that disrupted mitochondrial homeostasis impairs AM maturation and maintenance *in vivo* (Gao et al., 2022) (**Figure 1**). The mitochondrial transcription factor A (TFAM) is critical for mitochondrial transcription initiation and DNA replication. Specific knockout of TFAM in AMs impairs OXPHOS and mitochondrial fitness, and results in a dramatic reduction in the number of mature AMs. While TFAM deficiency does not influence AM development, it has a profound effect on their proliferative capacity, leading to accumulation of surfactants in the lung (Gao et al., 2022). Moreover, the lung environment also influences AM metabolism, as *ex vivo*-cultured AMs up-regulate expression of genes involved in glucose uptake and glycolysis, whereas peritoneal macrophages drastically reduce glucose uptake when intranasally transferred to the lung (Svedberg et al., 2019). These observations underpin the great impact of yet to be defined tissue signals or cues from the lung niche on AM mitochondrial metabolism and metabolic adaptations.

## Alveolar Macrophage Metabolism During Bacterial Infection

Much of our current knowledge regarding AM immunometabolism in the context of bacterial infection has resulted from studying *Mycobacterium tuberculosis* (Mtb) infection. Being the first host cells that Mtb encounters during infection, infected AMs promote Mtb dissemination to other innate immune cells by relocating from airways to the interstitium in an IL-1R-dependent manner (Cohen et al., 2018). Use of Mtb reporter strains reveals that AMs are a permissive cellular niche for Mtb growth during early stages of infection (Huang et al., 2018). Such permissiveness appears to be regulated by AM metabolism. Mtb-infected AMs increase fatty acid uptake, display higher OXPHOS, and engage in FAO, whereas monocyte-derived lung IMs that control Mtb growth exhibit greater glycolytic activity (Huang et al., 2018). Inhibition of FAO using etomoxir in BMDMs suppresses Mtb growth and is associated with a reduction in expression of IFN-β, a detrimental cytokine to Mtb (Mayer-Barber et al., 2014; Ji et al., 2019). These data agree with a separate study demonstrating the pivotal role of mitochondrial fatty acid metabolism in supporting type 1 interferon production by plasmacytoid dendritic cells (Wu et al., 2016). In addition, oxfenicine and trimetazidine, two FAO inhibitors, restrict Mtb growth in BMDMs similar to carnitine palmitoyltransferase 2 (Cpt2)-deficient BMDMs where FAO is prohibited (Chandra et al., 2020). Overall, both pharmacological and genetic data highlight the critical role of macrophage FAO in controlling Mtb, at least *in vitro*. While blockade of FAO in BMDMs has been proposed to promote recruitment of NADPH oxidase to autophagosomes to eliminate Mtb (Chandra et al., 2020), the function of AM FAO remains unclear during Mtb and other bacterial infections *in vivo*, including pathways that may fuel mitochondrial metabolism in AMs during infection.

Glycolysis is another major metabolic pathway that generates ATP and is typically elevated in pro-inflammatory macrophages during bacterial infection *via* pattern recognition receptor signaling. Glycolytic metabolism is required for activation, phagocytosis, and production of pro-inflammatory cytokines in M1 macrophages (O’neill and Pearce, 2016). However, whether AMs can engage in glycolysis *in vivo* is elusive. Murine AMs can secrete IL-6 and TNF-α upon LPS stimulation. Unlike M1 macrophages, such pro-inflammatory responses are not affected in glucose-free medium or when lactate dehydrogenase (LDH) is inhibited (Woods et al., 2020). On the other hand, human AMs skew metabolism toward OXPHOS with a high glycolytic capacity (Gleeson et al., 2018). Mtb-infected human AMs produce more lactate, the end-product of glycolysis, and display impaired control of Mtb growth in glucose deprivation or when blocking host LDH, indicating metabolic reprogramming toward glycolysis (Gleeson et al., 2016). Importantly, increased susceptibility to Mtb infection in smokers has been linked to the attenuated glycolytic metabolism in AMs (Gleeson et al., 2018). Moreover, extracellular flux analysis reveals Mtb infection impairs glycolysis and drives mitochondrial stress in AMs by inducing type 1 interferon (Olson et al., 2021). As the lung environment substantially impacts AM metabolism, *in vitro* and *ex vivo* analyses must be interpreted carefully. Notably, glycolysis in AMs can be beneficial for bacteria during pulmonary infections. For example, *Legionella pneumophila*, an intracellular pathogen that mainly targets AMs and neutrophils (Copenhaver et al., 2014), can secrete MitF effector, a GTPase activator to induce mitochondrial fission in human monocyte-derived macrophages. The fragmented mitochondria impair OXPHOS and ultimately lead to elevated glycolysis that favors *L. pneumophila* replication in macrophages (Escoll et al., 2017). However, these results merit further validations in AMs *in vivo*.

Mechanistically, several transcription factors have recently been linked to regulation of AM function by promoting metabolic reprogramming (**Figure 1**). Hypoxia-inducible factor-1α (HIF-1α) is a key oxygen sensor that controls gene expression to adapt cellular metabolism to hypoxia and inflammation. Stabilization of HIF-1α in AMs with dimethyloxalylglycine (DMOG) enhances expression of enzymes that promote glycolysis and lead to a marked reduction in OXPHOS (Woods et al., 2020). During Mtb infection, HIF-1α is required for the metabolic shift to glycolysis in macrophages and control of infection *in vivo* (Braverman et al., 2016; Braverman and Stanley, 2017). Harnessing HIF-1α using an iron chelator, desferrioxamine, supports functions of primary human macrophages during Mtb infection by promoting glycolysis that further boosts production of IL-1β and TNFα (Phelan et al., 2020). Additionally, exposing AMs to the HIF-1α stabilizer DMOG or the FAO inhibitor etomoxir induces lipid droplet formation in the presence of Mtb lipids (Genoula et al., 2020), consistent with another study demonstrating an indispensable role of HIF-1α in lipid droplet formation and maintenance in macrophages during Mtb infection (Knight et al., 2018). Furthermore, many pathogenic bacteria use virulence factors to target β-catenin signaling and promote growth and disease progression (Silva-Garcia et al., 2019). Traditionally, canonical β-catenin signaling is critical for cell growth and proliferation (Valenta et al., 2012). However, β-catenin can bind to HIF-1α which in turn promotes glycolysis and suppresses mitochondrial metabolism in AMs following influenza virus infection (Zhu et al., 2021). Therefore, this non-canonical β-catenin-HIF-1α axis functions as a fine-tuning regulator for balancing pro-inflammatory responses and self-renewal of AMs through metabolic reprogramming.

Nuclear factor erythroid 2-related factor 2 (Nrf2) is another transcription factor that serves as a key regulator of cellular metabolism (He et al., 2020; Ryan et al., 2022). Nrf2 can be activated by itaconate, one of the most abundant metabolites in LPS-stimulated macrophages, generated from cis-aconitate in the mitochondrial Krebs cycle through expression and activity of aconitate decarboxylase 1 (Acod1) (Mills et al., 2018). Activation of Nrf2 in macrophages has been postulated as an essential regulatory mechanism to limit inflammation during infection (Thimmulappa et al., 2006; Athale et al., 2012). Some bacterial species modulate Nrf2 activity to facilitate their own survival in AMs. *Coxiella burnetii* (*C. burnetii*), the causative agent of human Q fever, is well-known for using multiple strategies to perturb host cell signaling that facilitates growth in a lysosome-derived vacuole in macrophages. *C. burnetii* activates Nrf2 signaling by preventing its degradation and promoting translocation to the nucleus in human AMs (Winchell et al., 2018). Infected human AMs also increase production of transglutaminase 2 and shift to an M2-like phenotype, a preferred cellular niche for *C. burnetii* replication (Dragan et al., 2019). In addition, a newly identified *C*. *burnetii* effector protein, mitochondrial *Coxiella* effector protein C (MceC), interacts with components of the mitochondrial quality control machinery, highlighting the possibility of manipulating macrophage metabolism to facilitate intracellular growth (Fielden et al., 2021). Similarly, Mtb-infected AMs up-regulate an Nrf2-associated gene signature (Rothchild et al., 2019). Disruption of Nrf2 in myeloid cells and CD11c^+^ cells improves control of Mtb growth and activation of AMs in mice (Rothchild et al., 2019), in line with another study revealing that Nrf2 regulatory activity is mediated by blocking RNA polymerase II recruitment to inhibit pro-inflammatory cytokine transcription (Kobayashi et al., 2016). Moreover, proteomics and metabolomics analyses demonstrate that Nrf2 activation promotes mitochondrial fusion and reprogramming of the metabolic landscape in macrophages (Fielden et al., 2021). Lastly, activation of Nrf2 enhances nutrient uptake, OXPHOS, and glycolysis in myeloid-derived suppressor cells (MDSCs) and contributes to MDSC expansion (Ohl et al., 2018). Altogether, Nrf2 finetunes macrophage metabolism and likely bridges host cell metabolism and bacterial infection. It remains unknown the extent to which Nrf2 is required for bacterial infection-mediated metabolic reprogramming of AMs, and the specific metabolic pathways that Nrf2 targets during infection.

## Interstitial Macrophage Metabolism

Pulmonary IMs are monocyte-derived lung resident macrophages with great heterogeneity of phenotypes and functions. At steady state, at least two populations of IMs can be identified based on differential expression of Lyve1, CD206, MHCII, and CCR2 (Gibbings et al., 2017; Chakarov et al., 2019; Schyns et al., 2019; Dick et al., 2022). Interestingly, the anatomical niche harboring IMs subsets are distinct. Lyve1^low^MHCII^hi^ IMs are associated with nerves, whereas Lyve1^hi^MHCII^low^ IMs are near blood vessels, further indicating divergent functions (Chakarov et al., 2019). Unlike AMs, the IM metabolism is much less understood. Recently, expression of folate receptor 2 (Folr2) has been shown in a subset of IMs across organs, including the lung, highlighting the metabolic need for folic acid in these cells (Dick et al., 2022). The folate cycle belongs to one-carbon metabolism that provides methyl groups for methylation reactions, synthesis of DNA, amino acids, and phospholipids (Ducker and Rabinowitz, 2017). While the role of one-carbon metabolism is not fully elucidated in innate immune cells, a recent study demonstrated a pivotal role for serine biosynthesis, part of one-carbon metabolism, in promoting M2 macrophage activation (Raines et al., 2022). Inhibition of phosphoglycerate dehydrogenase (Phgdh) or knockout of phosphoserine aminotransferase 1 (Psat1), two key enzymes in serine biosynthesis, attenuates mitochondrial metabolism and JMJD3-mediated histone demethylation to suppress immunosuppressive M2 polarization in macrophages (Raines et al., 2022). Moreover, increased Folr2 expression is associated with M2 macrophage activation in tumors (Puig-Kroger et al., 2009), consistent with the Folr2^+^ lung IM subset displaying increased CD206 expression (Dick et al., 2022). Altogether, these studies highlight a previously underappreciated metabolic pathway that regulates macrophage function and merits further investigation of its role in lung IMs.

During early stages of Mtb infection, compared to AMs, IMs are less permissive for Mtb, which is likely attributed to increased glycolytic activity (Huang et al., 2018) (**Figure 1**). IMs from Mtb-infected mice produce more lactic acid than AMs and exhibit impaired production of IL-1β and TNFα upon glycolysis inhibition, recapitulating metabolic signatures described in M1 macrophages (O’neill and Pearce, 2016; Huang et al., 2018). However, IMs can incorporate and use exogenous long chain fatty acids, indicating active mitochondrial metabolism (Huang et al., 2018). Indeed, stimulating IMs with bacterial CpG leads to IM expansion and elevated production of IL-10 (Sabatel et al., 2017), a cytokine that can eliminate dysfunctional mitochondria by autophagy and maintain mitochondrial integrity by suppressing mTOR signaling in LPS-stimulated macrophages (Ip et al., 2017). These data further underpin the essential role of mitochondrial metabolism in IMs. Finally, mitochondrial respiration in IMs can be specifically regulated by β-catenin signaling activated by the endothelial cell-derived Wnt molecule Rspondin3 in the LPS-induced lung injury model (Zhou et al., 2020). While these data highlight active crosstalk between IMs and structural cells in the lung, whether this mechanism is triggered during bacterial infection needs further examination. Altogether, IM metabolism is context-specific and highly plastic. Given their heterogeneous nature, diverse metabolic profiles may reflect distinct metabolic signatures in different IM subsets.

## Trained Immunity in Lung Macrophages

**Figure 2:**
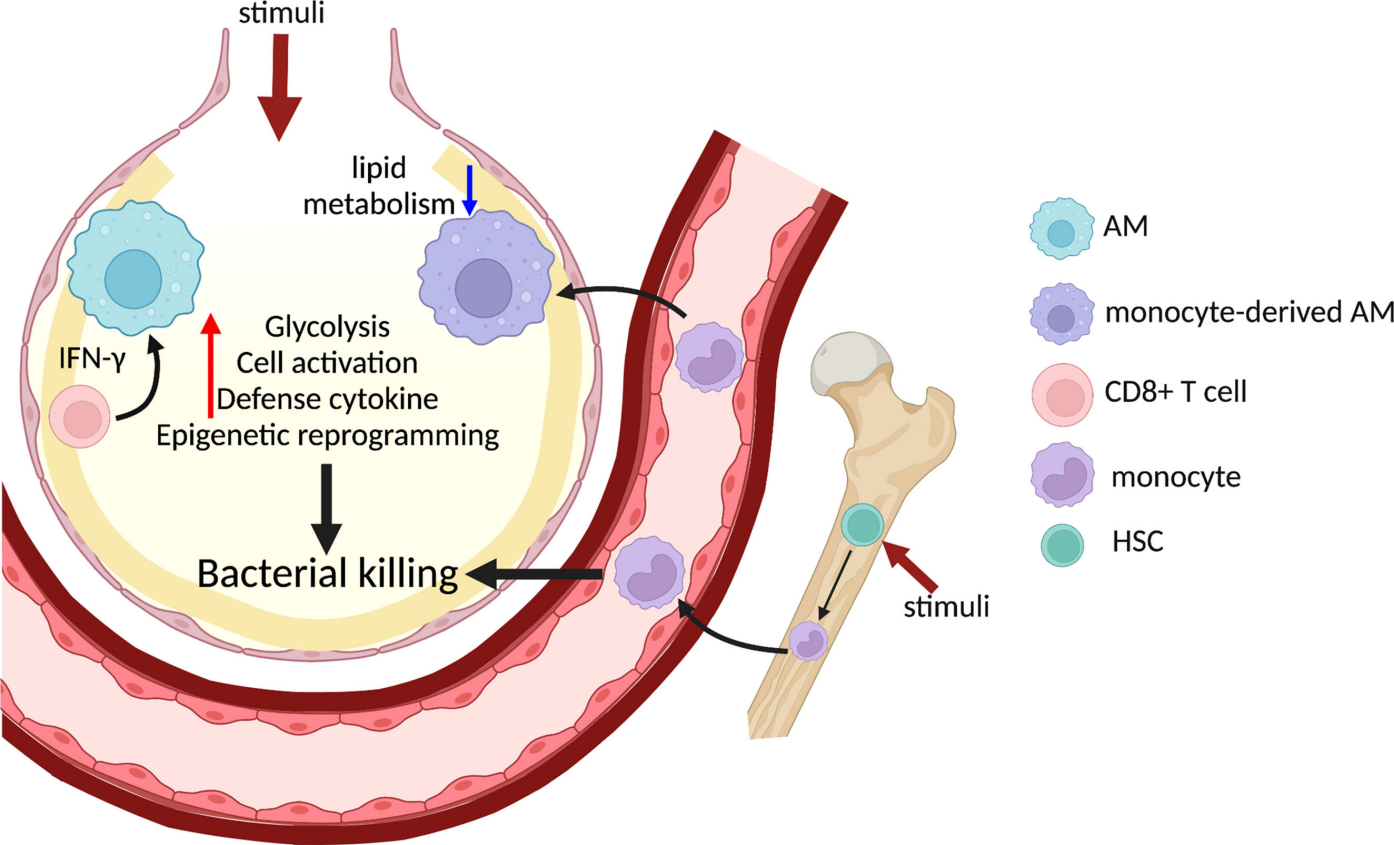
Trained immunity in lung macrophages promotes host defense against bacterial infection. Systemic administration of BCG or β-glucan activates HSC and induces myelopoiesis in the bone marrow and generates trained monocytes/macrophages. Direct administration of various stimuli to the alveolar space also triggers trained immunity in AMs, which is dependent on IFNγ from CD8^+^ T cells. Monocytes are recruited to the inflamed lung and differentiate into monocyte-derived AMs, a population which displays reduced lipid metabolism and enhanced bacterial killing. Trained lung macrophages alter chromatin accessibility and undergo extensive metabolic rewiring, which result in elevated glycolysis and cytokine production to further promote bacterial clearance in the lung. HSC, homeostatic stem cell.

Trained immunity is an emerging field that integrates cellular functionality, metabolism, and epigenetic regulation of macrophages. This version of immunity consists of long-term functional reprogramming of innate immune cells, particularly macrophages, that leads to a more robust, yet non-specific, response to secondary insult. Altered functionality of trained macrophages is now believed to be mediated by metabolic reprogramming and sustained changes in epigenetic modifications (Netea et al., 2020). Initially, many studies in this field were performed *in vitro*. However, recent studies using *in vivo* models demonstrate that trained immunity can be induced at multiple levels, including in the bone marrow and in resident lung macrophages (**Figure 2**). For example, intravenous injection of Bacillus Calmette-Guérin (BCG) leads to transcriptional profile changes in hematopoietic stem cells that induce an enhanced myelopoiesis in the bone marrow (Kaufmann et al., 2018). BCG injection elicits stronger protective responses against Mtb infection in a monocyte-dependent manner, suggesting pre-programmed alteration of monocyte precursors in the bone marrow upon BCG stimulation (Kaufmann et al., 2018). Intraperitoneal injection of mice with β-glucan also induces a comparable response in the bone marrow and promotes a protective response against Mtb infection (Moorlag et al., 2020). It is still unclear whether systemic administration of BCG or β-glucan induces trained immunity in tissue resident cells at specific mucosal sites. In a separate study, AMs reprogrammed by inoculation of an adenoviral vaccine exhibited sustained protection against *Streptococcus pneumoniae* infection (Yao et al., 2018). Trained AMs display enhanced activation, cytokine production, and glycolysis that relies on CD8^+^ T cell-derived IFNγ, underscoring a cooperative network between trained and adaptive immunity (Yao et al., 2018). Moreover, not all inflammatory stimulation triggers development of trained immunity in AMs. Primary pneumonia induced by bacteria or influenza virus can induce AM paralysis characterized by significantly reduced phagocytosis upon secondary challenge (Roquilly et al., 2020). AM paralysis is associated with altered histone H3 Lys27 acetylation in NF-κB-regulated genes (Roquilly et al., 2020). Interestingly, paralyzed AMs still produce lactate upon LPS stimulation, indicating active glycolysis (Roquilly et al., 2020). While the specific metabolic pathways that mediate these responses remain unclear, mitohormesis triggered by TLR-dependent mitochondrial ROS and reactive electrophilic species may promote macrophage tolerance (Timblin et al., 2021). Additionally, whether trained immunity confers protection against infection by inducing alteration of macrophage ontogeny merits further investigation. Influenza infection induces generation of persistent CCR2^+^ monocyte-derived AMs that exhibit chromatin accessibility and transcriptional profiles distinct from resident AMs (Aegerter et al., 2020). Importantly, monocyte-derived AMs have reduced lipid metabolism and are more protective against secondary bacterial infection, although this protective signature wanes over time, suggesting re-adaptation of macrophages to the lung environment (Aegerter et al., 2020). This study highlights the importance of macrophage origin as a novel determining factor of trained immunity.

## Discussion

The location of lung macrophages warrants unique phenotypic and metabolic heterogeneity when encountering pulmonary bacteria, which provide numerous potential targets for therapeutics and novel vaccine strategies. However, many remaining questions need to be addressed in this exciting new field. One should be to interrogate crosstalk between lung macrophages and structural cells during bacterial infection. A recent study demonstrated the central role of the alveolar epithelium in communicating with lung immune cells across the airway and enhancing glycolysis-dependent inflammation in monocytes to promote antibacterial defense (Liu et al., 2020), implicating the impact of extrinsic signaling of non-immune cells on lung macrophage metabolism. Moreover, our current method for lung macrophage extraction may have a substantial impact on metabolism and also lead to a loss of spatial location. Indeed, use of Mtb reporter strains reveals differential replication of bacteria at the core and cuff of lung granulomas (Lavin and Tan, 2022), which demonstrates the importance of considering spatial relationships of host-bacteria interactions and the possibility of using bacterial reporters to probe lung macrophage metabolism *in situ* (Macgilvary and Tan, 2018). Finally, one of the most important perspectives is to translate our discoveries into human treatments. We must better understand underlying metabolic regulation of human lung macrophages during bacterial infection using novel platforms such as primary human lung macrophages or human precision-cut lung slices (hPCLS) (Dragan and Voth, 2021). Integration of these primary tissue platforms, reporter bacterial strains, and metabolic analyses will no doubt greatly advance understanding of lung macrophage metabolism and provide insight on how to manipulate lung macrophage metabolism for treating pulmonary bacterial infection.

## Author Contributions

All authors listed have made a substantial, direct, and intellectual contribution to the work and approved it for publication.

## Funding

DV is supported by NIH grants AI144506 and P20GM103625 through the Center for Microbial Pathogenesis and Host Inflammatory Responses at UAMS. SH is funded by Andrew McDonough B+ Foundation Childhood Cancer Research Grant Award, Cancer Research Institute CLIP Investigator Award, the VeloSano Pilot Award, Case Comprehensive Cancer Center American Cancer Society Pilot Grants (IRG-91-022-19, IRG-16-186-21), Case GI SPORE DRP Grant (5P50CA150964-08), and the Cleveland Digestive Research Core Center Pilot Grant (1P30DK097948). LH is supported by NIH grant P20GM103625 through the Center for Microbial Pathogenesis and Host Inflammatory Responses at UAMS, American Lung Association grant CA-828143, Arkansas Bioscience Institute, Sturgis Foundation as well as Start-up package from College of Medicine at UAMS.

## Conflict of Interest

The authors declare that the research was conducted in the absence of any commercial or financial relationships that could be construed as a potential conflict of interest.

## Publisher’s Note

All claims expressed in this article are solely those of the authors and do not necessarily represent those of their affiliated organizations, or those of the publisher, the editors and the reviewers. Any product that may be evaluated in this article, or claim that may be made by its manufacturer, is not guaranteed or endorsed by the publisher.
